# Mechanical Performance and Microstructural Evolution of Rotary Friction Welding of Acrylonitrile Butadiene Styrene and Polycarbonate Rods

**DOI:** 10.3390/ma16093295

**Published:** 2023-04-22

**Authors:** Chil-Chyuan Kuo, Naruboyana Gurumurthy, Hong-Wei Chen, Song-Hua Hunag

**Affiliations:** 1 Department of Mechanical Engineering, Ming Chi University of Technology, No. 84, Gungjuan Road, New Taipei City 243, Taiwan; 2 Research Center for Intelligent Medical Devices, Ming Chi University of Technology, No. 84, Gungjuan Road, New Taipei City 243, Taiwan; 3 Department of Mechanical Engineering, Chang Gung University, No.259, Wenhua 1st Rd., Guishan Dist., Taoyuan City 333, Taiwan; 4 Department of Mechanical Engineering, Presidency University, Rajankunte, Near Yelhanka, Bangalore 700073, India; 5 Li-Yin Technology Co., Ltd., No. 37, Lane 151, Section 1, Zhongxing Road, Wugu District, New Taipei City 241, Taiwan

**Keywords:** solid-state welding, polymer friction welding, polymer welded joints, RFW, dissimilar polymer welds

## Abstract

Rotary friction welding (RFW) is a green manufacturing technology with environmental pollution in the field of joining methods. In practice, the welding quality of the friction-welded parts was affected by the peak temperature in the weld joint during the RFW of dissimilar plastic rods. In industry, polycarbonate (PC) and acrylonitrile butadiene styrene (ABS) are two commonly used plastics in consumer products. In this study, the COMSOL multiphysics software was employed to estimate the peak temperature in the weld joint during the RFW of PC and ABS rods. After RFW, the mechanical performance and microstructural evolution of friction-welded parts were investigated experimentally. The average Shore A surface hardness, flexural strength, and impact energy are directly proportional to the rotation speed of the RFW. The quality of RFW is excellent, since the welding strength in the weld joint is better than that of the ABS base materials. The fracture occurs in the ABS rods since their brittleness is higher than that of the PC rods. The average percentage error of predicting the peak temperature using COMSOL software using a mesh element count of 875,688 for five different rotation speeds is about 16.6%. The differential scanning calorimetry curve for the friction-welded parts welded at a rotation speed of 1350 rpm shows an endothermic peak between 400 to 440 °C and an exothermic peak between 600 to 700 °C, showing that the friction-welded parts have better mechanical properties.

## 1. Introduction

Rotary friction welding (RFW) [1] is one of the approaches of friction welding that is considered solid-state welding, which has lower energy consumption and environmental impact compared to gas metal arc welding. The process of RFW is that one welded element is rotated at a constant speed while the other welded element remains stationary under an axial force. In general, RFW gives many features based on its practical experience in industry compared with adhesive bonding [2,3]. The RFW process provides a lower peak temperature in the weld joint compared with fusion welding (FW) [4,5,6]. Therefore, a wide range of similar or dissimilar materials can be joined efficiently and economically. The products obtained by RFW have low defect rates and low distortion. In addition, the manufacturing costs can be reduced significantly compared with the subtractive technique, such as milling machining from buck materials [7,8].

Eslami et al. [9] reviewed the friction stir welding tooling for polymers and analyzed the weld strengths for different polymeric materials. Paoletti et al. [10] investigated the forces and temperatures in the friction spot stir welding of thermoplastic polymers. The results showed that the increase in the tool rotational speed will reduce the processing forces. Lambiase et al. [11] investigated the influence of the plunging force in the friction stir welding of polycarbonate sheets on the mechanical behavior of the welds. The results revealed that the mechanical behavior of the welds can be improved up to 37 % by plunging force. The shear strength of 34.5 MPa that yields the base material can be obtained by optimal conditions. Rehman et al. [12] investigated the effects of preheating on joint quality in the friction stir welding of polyethylene. The results showed that proper welding of this bimodal high-density polyethylene takes place when the material is maintained at high temperatures. Large elongations in the order of 60% and weld efficiencies in excess of 100% were also achieved by optimal welding temperatures. Skowrońska et al. [13] assessed the structural properties of friction-welded joints. The results revealed that a surface hardness above 340 HV was obtained in the weld joint. Dhooge et al. [14] proposed a new variant friction-welding process for the fully automatic joining of pipelines. Optimization of the duration of the friction phase of the friction-welding process was also investigated. Anwar et al. [15] investigated the microstructure, mechanical properties, and grain size of the alloy after RFW. The minimum grain size was successfully met by postweld heat treatment with improved elongation and strength. It was found that the weld metal grain size of the postweld heat treatment joints is about 35 ± 4 µm. The average grain size of the weld metal in the as-welded condition is about 20 ± 2 µm, in contrast to the weld metal grain size of the PWHT joints. Ishraq et al. [16] investigated the weld strength by optimizing the welding process parameters. The results showed that the major reason for the high strength of a welded material used is the optimal level of fiberglass. Hangai et al. [17] studied the effects of the porosity of aluminum foam on the weldability to a polycarbonate plate. A welding strength of polycarbonate plate and Al foam higher than that of the base Al foam with a porosity of 80% can be obtained. Skowrońska et al. [18] investigated the microstructure of a friction-welded joint made of stainless steel with an ultrafine-grained structure made by hydrostatic extrusion. It was found that strength is the criterion for assessing the properties of the joint because of the complexity of the microstructure of the friction-welded joint. Zhang et al. [19] investigated a thermal compression bonding process in friction welding. It was found that the frictional flow greatly enhanced the formation of intermetallic compounds along the weld interface. Eliseev et al. [20] investigated the microstructural evolution in the transfer layer of aluminum alloy welds. The results showed that the size of the incoherent intermetallic particles and the volume fraction decreased towards to the center of the layer. Ma et al. [21] investigated the effects of temperature on mechanical performances of friction-stir-welded aluminum alloy joints. It was found that the reduction in the gradient along the thickness due to the pinhole increased heat input and material flow at the bottom.

Polymers are frequently used in some structures, because the major difference between metal and polymers is that polymers are lighter than metal. Acrylonitrile butadiene styrene (ABS) [22] and polycarbonate (PC) [23] are two commonly used plastics in consumer products. ABS is an thermoplastic engineering material that has high tensile strength and high resistance to chemical corrosion and physical impacts. In addition, ABS is easy to use in the injection-molding process since it has low melting point. Therefore, ABS plastic is suitable for making consumer products to withstand heavy use. PC plastic is considered as an engineering plastic since it has very good heat resistance, and is widely employed for more robust materials. However, hitherto little is known about the domain knowledge of the RFW of ABS and PC polymer rods. For this reason, the objective of this study is to establish domain knowledge of the RFW of ABS and PC rods. The RFW experiment was performed using a turning machine. During RFW, the peak temperature in the weld joint was determined using an infrared thermal imager. The COMSOL multiphysics software [24,25,26] was also employed to predict the peak temperature in the weld joint and to compare the results obtained by the experiment. After RFW, the mechanical properties and microstructural evolution of the friction-welded parts were characterized using Shore A hardness, three-point bending, and impact tests. Finally, domain knowledge of the RFW of ABS and PC rods was proposed. The melting behavior, solidification characteristics, and glass transition temperature of friction-welded joints was also conducted with differential scanning calorimetry (DSC).

## 2. Experimental Details

**Figure 1:**
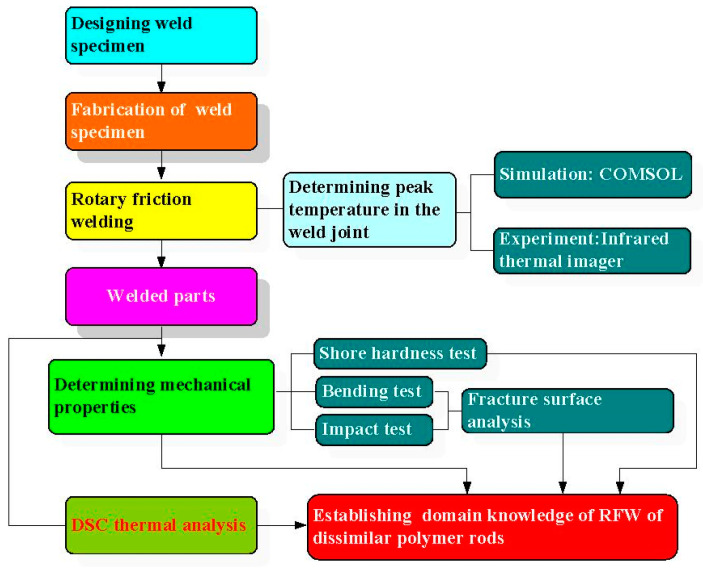
Flowchart of experimental details.

**Figure 2:**
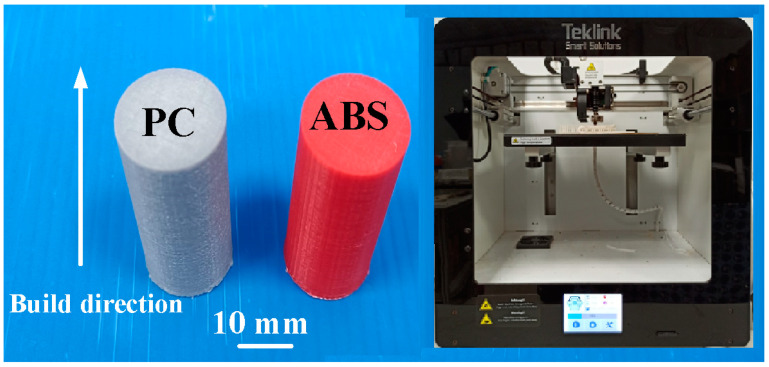
Two dissimilar workpieces for RFW in this study.

Figure 1 shows the flowchart of experimental details. The entire process includes designing weld specimen, fabrication of weld specimen, rotary friction welding, determining peak temperature in the weld joint via simulation by COMSOL, and experiment using infrared thermal imager, determining mechanical properties, Shore hardness tests, bending tests, impact tests, fracture surface analysis, DSC thermal analysis, and finally establishing domain knowledge of RFW of dissimilar polymer rods. In the simulation by COMSOL, the entire process involves thermal pattern analysis and suitable boundary conditions for the rotary friction model. At first, a COMSOL model is established, which involves identifying the components of the system and their physical properties. Then, the next step includes setting the parameters for RFW. Then, suitable boundary conditions, such as heat flux at various locations or temperature, need to be selected based on the system geometry and heat transfer mechanisms. Finally, peak temperature in the weld joint through the heat transfer needs to be identified, using heat transfer mechanisms such as conduction, convection, and radiation, to analyze the thermal behavior. The workpiece is a cylindrical rod with a diameter of 20 mm and a length of 40 mm. The welding workpieces were printed with a three-dimensional printing apparatus named fused deposition modeling (FDM), using two different thermoplastic filaments, i.e., PC (Thunder 3D Inc., New Taipei City, Taiwan) and ABS (Thunder 3D Inc., New Taipei City, Taiwan) [27]. Figure 2 shows the two dissimilar workpieces for RFW in this study. The FDM process parameters for ABS rods include infill percentage of 70%, print bed temperature at 100 °C, print speed of 80 mm/s, print temperature of 230 °C, shell thickness of 0.4 mm, and layer thickness of 0.1 mm. The FDM process parameters for PC rods involve infill percentage of 100%, print bed temperature at 100 °C, print speed of 80 mm/s, print temperature of 245 °C, shell thickness of 0.4 mm, and layer thickness of 0.1 mm. The printing strategy for the cylindrical polymer rod of ABS and PC is that the extruder moves in straight lines back and forth to create the parallel lines of the material.

**Figure 3:**
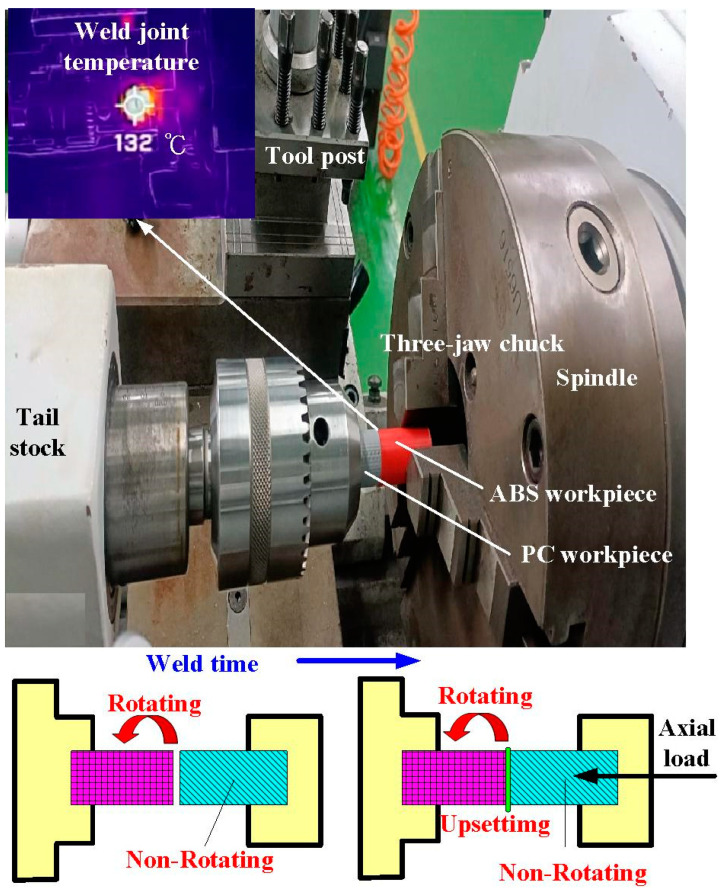
Situation of RFW.

**Figure 4:**
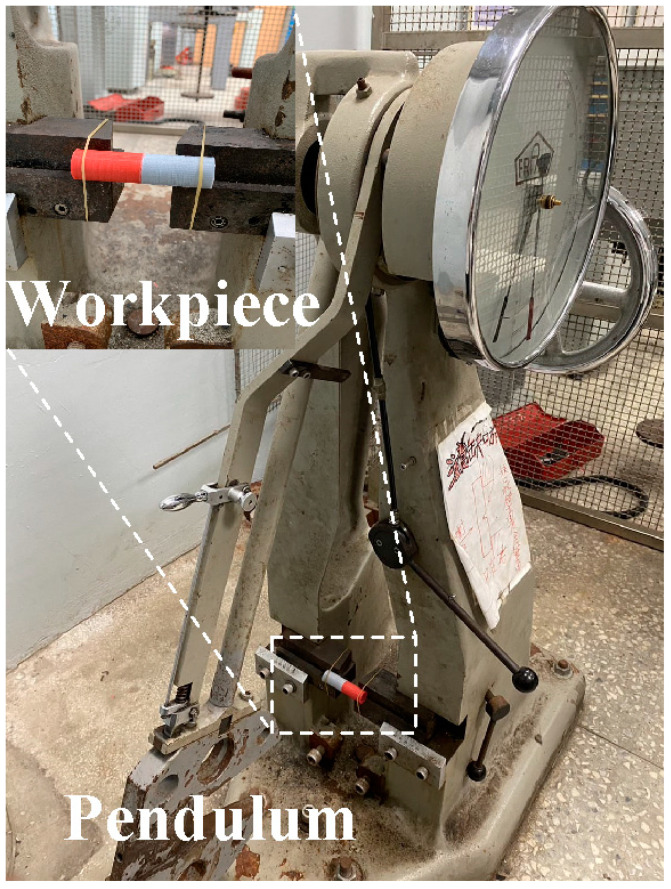
Experimental set-up for impact test.

**Figure 5:**
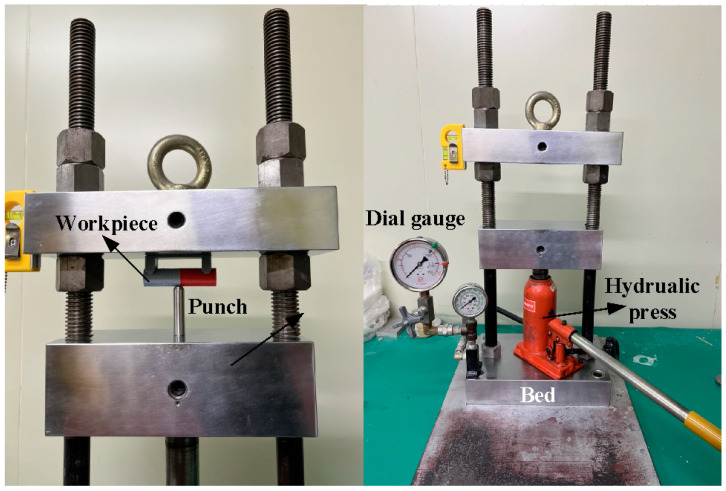
Experimental set-up for bending test.

Figure 3 shows the situation of RFW. A turning machine was selected as a friction welder to carry out RFW of dissimilar polymer rods. The welding time of RFW was set to 60 s, involving friction time of 30 s, forge time of 20 s, and cooling time of 10 s. To study the effects of rotation speed on the peak temperature in the weld joint, five rotation speeds, i.e., 330 rpm, 490 rpm, 650 rpm, 950 rpm, and 1350 rpm, were performed. During RFW, the peak temperature in the weld joint was recorded using an infrared camera (BI-TM-F01P, Panrico trading Inc., New Taipei City, Taiwan). After RFW, Charpy impact test (780, Instron Inc., Norwood, MA, USA), Shore A surface hardness test (MET-HG-A, SEAT Inc. New Taipei City, Taiwan), and bending test (RH-30, Shimadzu Inc., Kyoto, Japan) were performed to investigate the microstructural evolution and mechanical properties of the friction-welded parts. Figure 4 shows the experimental set-up for impact test. The insert is the close-up view showing the arrangement of the friction-welded parts in the impact test. Figure 5 shows the experimental set-up for bending test, showing the setting of the friction-welded parts during the bending test. The macrostructure of the fracture surfaces after bending and impact tests was performed by a field-emission scanning electron microscope (FE-SEM) (JEC3000-FC, JEOL Inc., Tokyo, Japan) and a stereo optical microscope (Quick Vision, Mitutoyo Inc, Tokyo, Japan). In this work, DSC (STA 409 PC Luxx Simultaneous thermal analyzer, Netzsch-Geratebau GmbH Inc., Waldkraiburg, Germany) analysis was used to calculate the melting and mesomorphic transitions along with their enthalpy and entropy of the friction-welded joints after RFW with five rotation speeds. The DSC experimental setup provides two separate crucibles for heating and cooling. One is for reference and another is for a sample to be investigated. DSC evaluation was carried out under controlled experimental conditions of continuous heating rate of 15 °C/min and continuous cooling rate of 15 °C/min in temperatures ranging from 50 °C to 250 °C. Thermal properties were investigated by two continuous exothermic cycles and an endothermic cycle at 25 mL/min of nitrogen gas supply.

## 3. Results and Discussion

**Figure 6:**
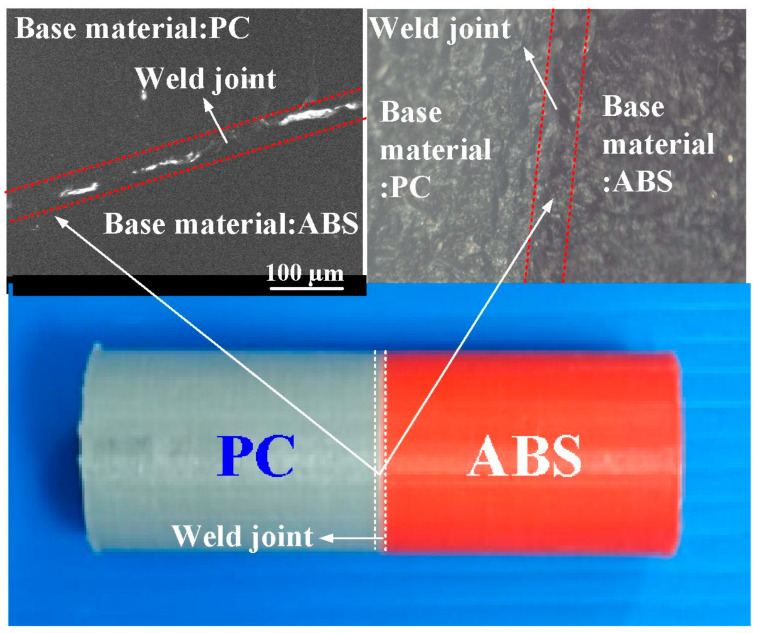
Friction-welded PC and ABS rods using RFW.

**Figure 7:**
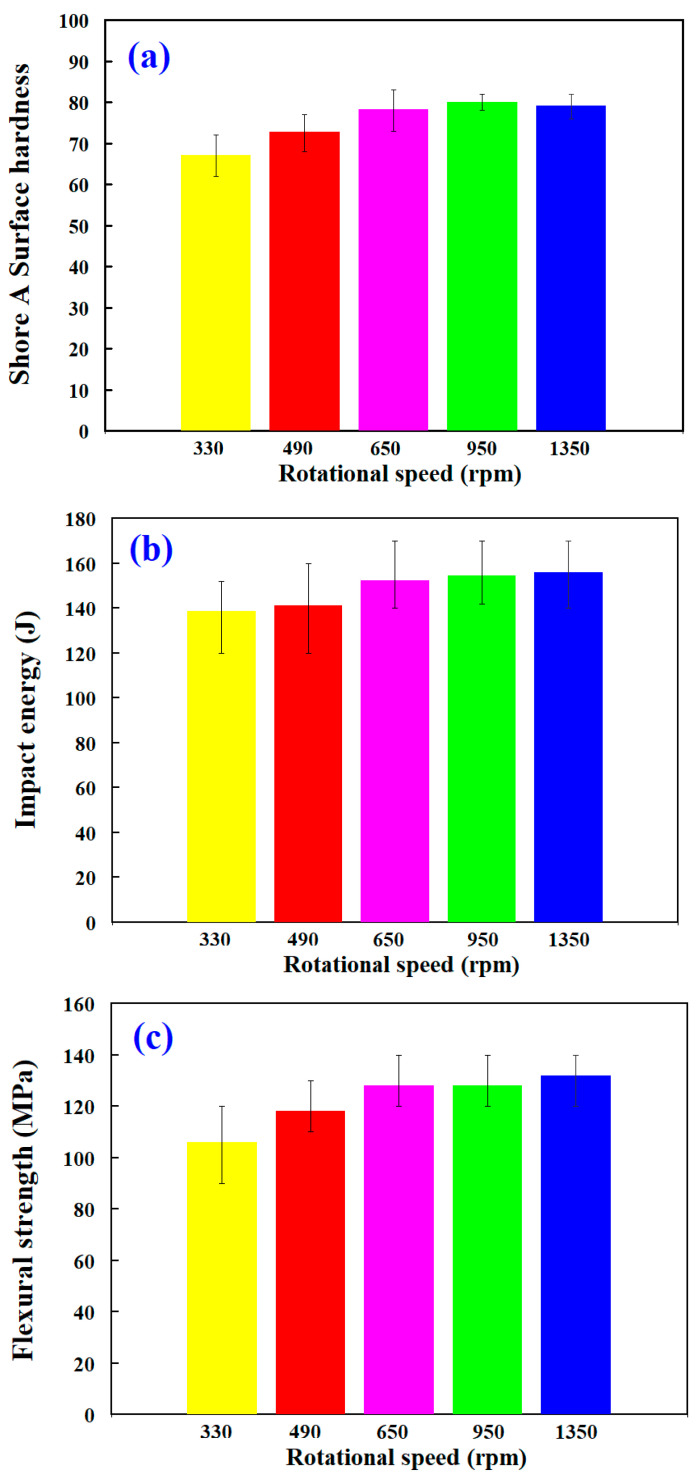
Mechanical properties of (**a**) surface hardness, (**b**) impact energy, and (**c**) flexural strength in the weld joint for RFW of dissimilar polymer rods under five rotation speeds.

Figure 6 describes the friction-welded PC and ABS rods using RFW. The top of this figure shows panoramic SEM micrographs of the friction-welded joint. This result reveals that the RFW of PC and ABS is acceptable since the bead width is consistent. Figure 7 shows the mechanical properties of surface hardness, impact energy, and flexural strength in the weld joint for RFW of dissimilar polymer rods under five rotation speeds. The maximum joint strength of 132 MPa, Shore A hardness of 80, and impact energy of 156 J can be obtained at a rotation speed of 1350 rpm. As can be seen, the increase in joint strength and surface hardness with increasing rotation speed is observed. This result reveals that the surface hardness, flexural strength, and impact energy are directly proportional to the rotation speed of RFW [28].

**Figure 8:**
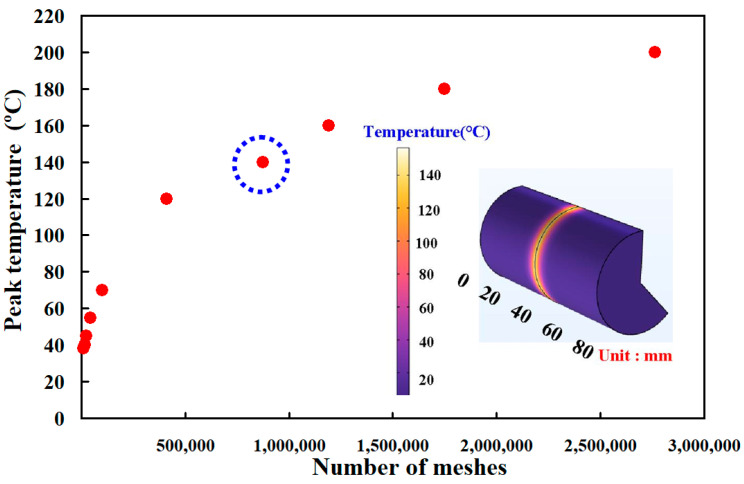
Number of meshes as a function of peak temperature in the weld joint.

Figure 8 shows the number of meshes as a function of peak temperature in the weld joint. Ten different kinds of mesh sizes, i.e., 0.4 mm, 0.5 mm, 0.6 mm, 0.7 mm, 0.8 mm, 0.9 mm, 1.0 mm, 1.1 mm, 1.2 mm, and 1.3 mm, were used to investigate the peak temperature in the weld joint during RFW. The insert shows the thermal model of RFW. It should be noted that a higher number of meshes provides more computation time. As can be seen, the peak temperature predicted by the COMSOL multiphysics software using a mesh element count of 875,688 is very close to that obtained by the experimental result. The mesh size is about 0.7 mm. This shows that a mesh element count of 875,688 is suitable for predicting the peak temperature in the RFW of PC and ABS rods.

**Figure 9:**
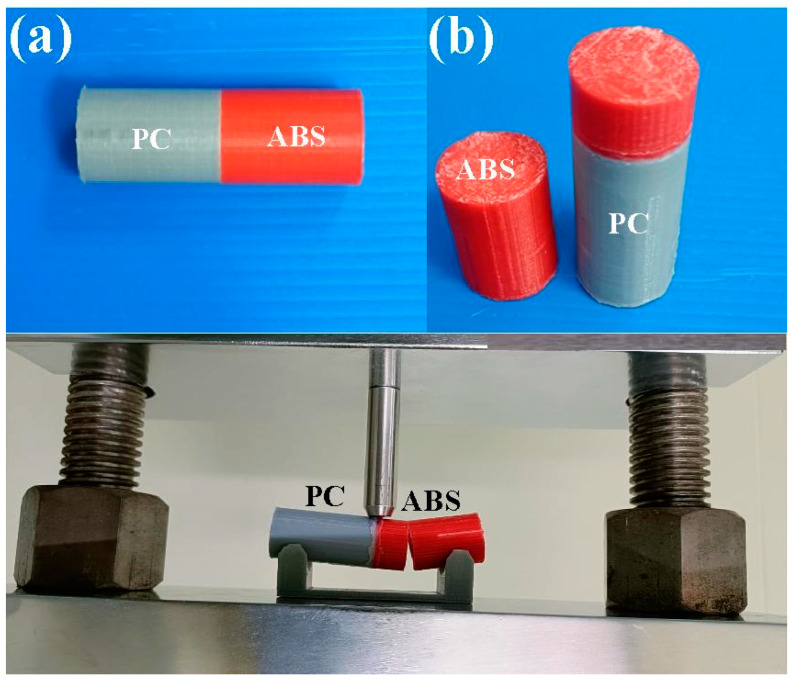
Friction-welded parts (**a**) before and (**b**) after bending tests.

**Figure 10:**
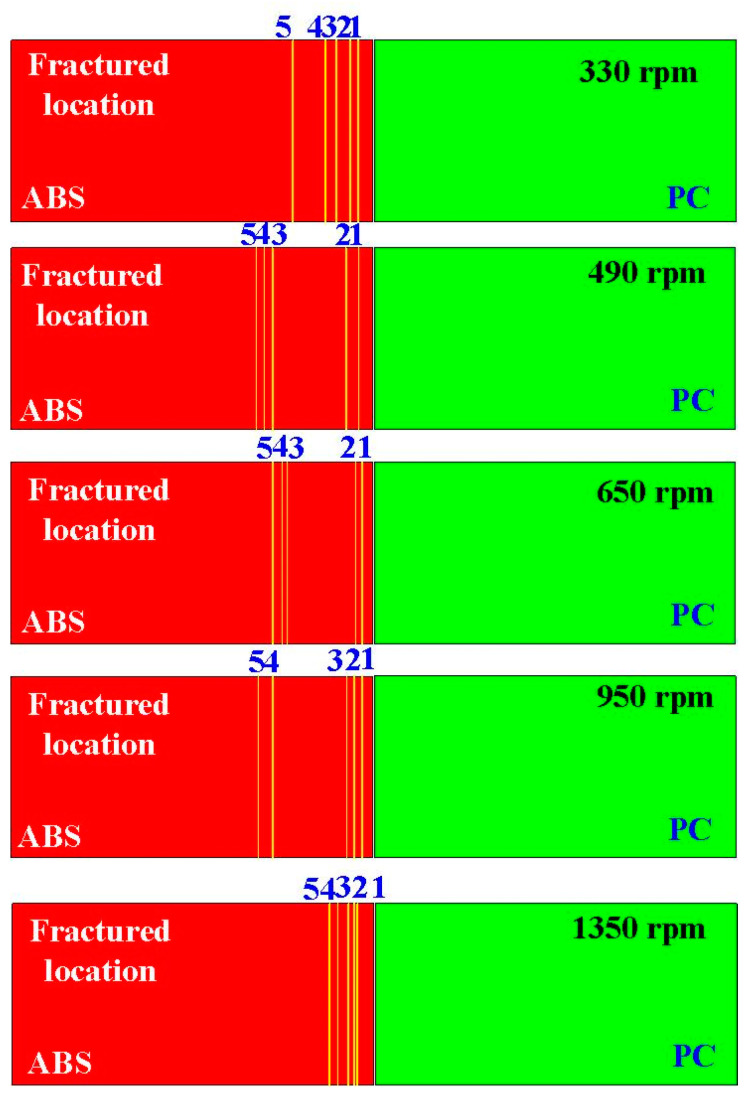
Fractured locations in the ABS rods after bending tests.

Figure 9 shows the friction-welded parts before and after bending tests. It was observed that the joining of ABS and PC by FRW is expected to improve the basic mechanical properties of the single ABS thermoplastic material. It is well known that the flexural strength of the PC material is better than that of the ABS material. According to the experimental results, the average flexural strength of the weld joint and the PC and ABS rods is about 132 MPa, 180 MPa, and 110 MPa, respectively. The fracture initiated in the ABS rods shows that the welding quality of FRW is excellent, since the welding strength in the weld joint is better than that of the ABS base materials [29]. In general, the ABS polymer is a relatively soft and flexible material. The PC polymer has a higher glass transition temperature than the ABS polymer, showing it can withstand higher temperatures before it starts to soften and lose its shape. Therefore, the PC polymer seems to be a better choice for parts that need to operate in high-temperature environments. In particular, the fractured locations in the ABS rods after bending tests are random, as shown in Figure 10.

**Figure 11:**
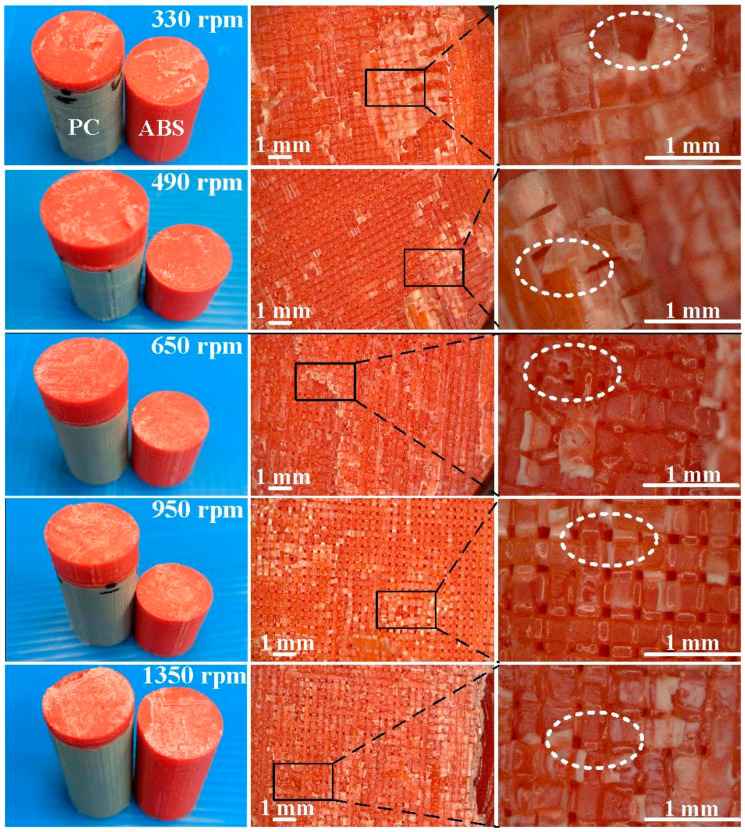
Fracture surfaces of ABS after three-point bending tests for six different rotation speeds.

**Figure 12:**
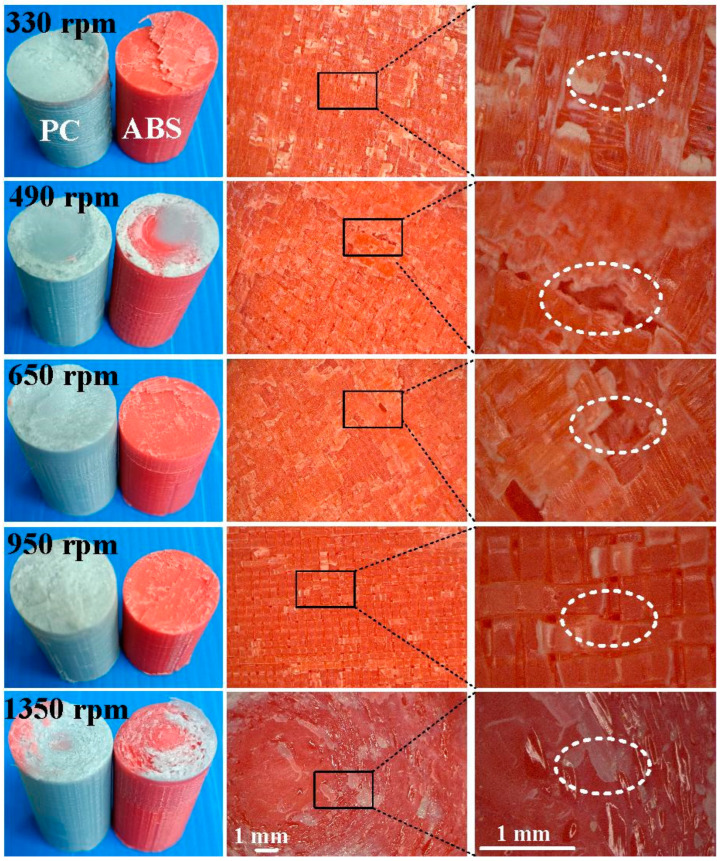
Fracture surfaces of ABS after impact tests for six different rotation speeds.

**Figure 13:**
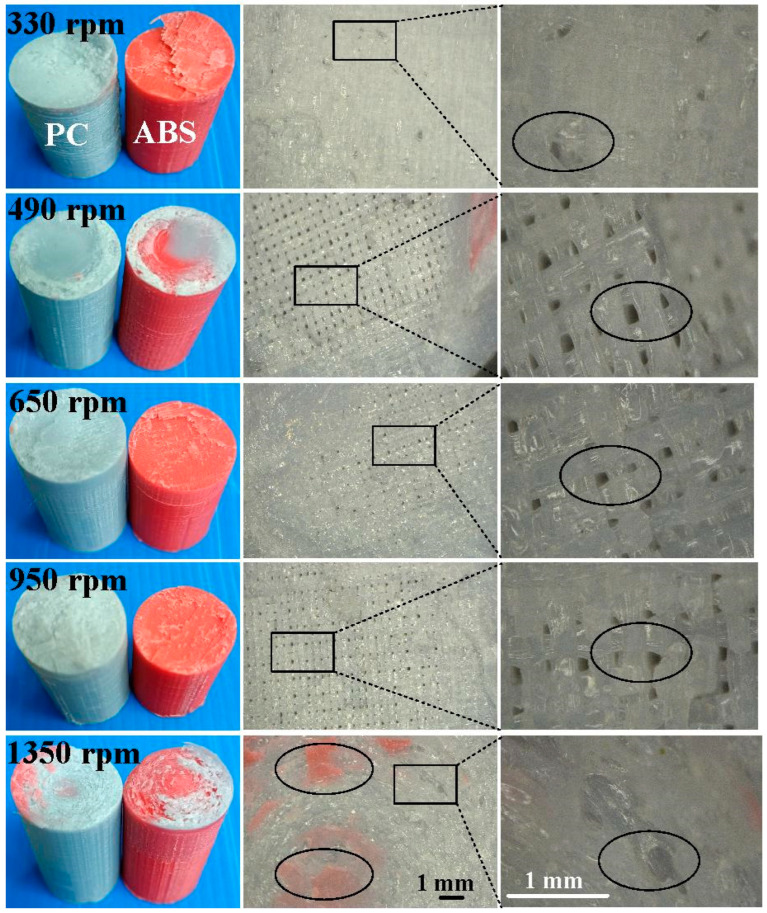
Fracture surfaces of PC after impact tests for six different rotation speeds.

Figure 11 shows the fracture surfaces of ABS after three-point bending tests for six different rotation speeds. Figure 12 describes the fracture surfaces of ABS after impact tests for six different rotation speeds. A small region of porous surface was also observed and highlighted. The porous surface is caused by insufficient molecular diffusion and crystallinity in FDM printing, and can be reduced by microwave [30]. Figure 13 describes the fracture surfaces of PC after impact tests for six different rotation speeds. The formation of voids and cracks after the impact test can be identified by analyzing the surfaces of fractured parts. As can be seen, jagged and irregular surfaces were found. According to the optical microscopic images of fractured surfaces, two phenomena are found. One is that the ABS material exhibits cracked surface textures. The other is that the PC material exhibits a porous structure.

**Figure 14:**
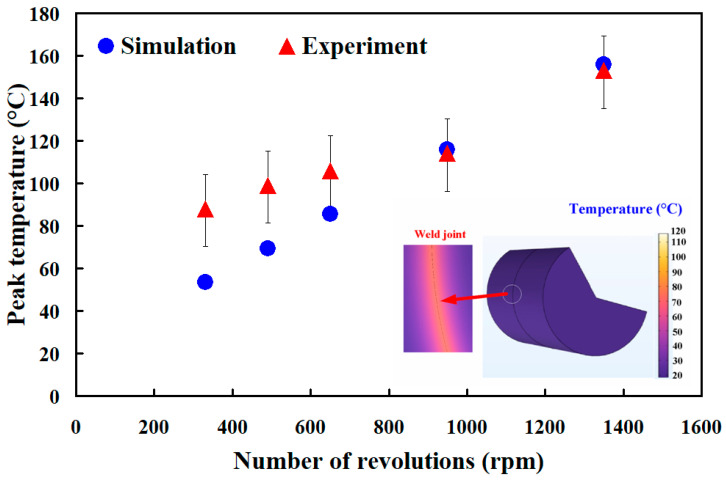
Comparison of the experimental and numerical simulation results of the peak temperature for RFW of PC and ABS rods at five different rotation speeds.

**Figure 15:**
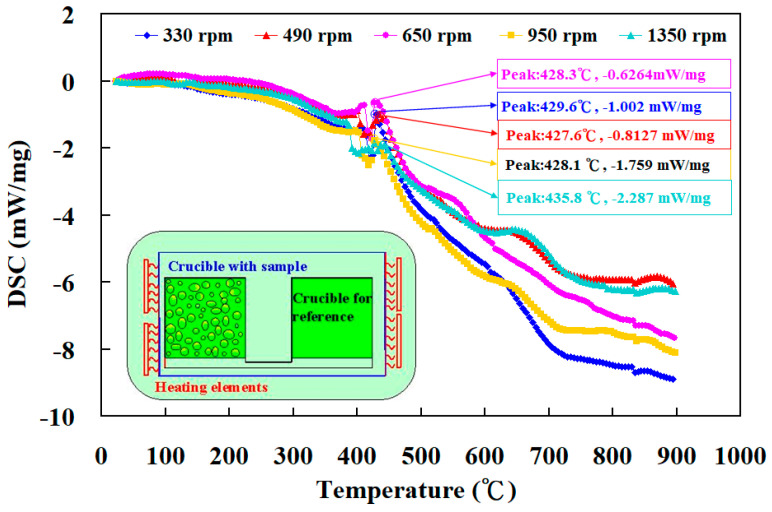
DSC curve comparisons for friction-welded part under five rotation speeds.

Figure 14 shows the comparison of the experimental and numerical simulation results of the peak temperature for the RFW of PC and ABS rods at five different rotation speeds. As can be seen, the percentage error of the peak temperature between the experimental and numerical simulation results for 330 rpm, 490 rpm, 650 rpm, 950 rpm, and 1350 rpm is about 38.6 %, 29.3%, 18.9%, −1.8%, −2.0%, and 16.6%, respectively. Therefore, the average percentage error of predicting the peak temperature using COMSOL software for five different rotation speeds is about 16.6%. In general, DSC analyzes the melting behavior, solidification characteristics, and glass transition temperature. Figure 15 shows the DSC curve comparisons for the friction-welded part under five rotation speeds. The insert shows the DSC setup for the samples fabricated by five rotation speeds. As can be seen, the DSC peak appears at a temperature of 429 °C, showing that there is a significant thermal event happening in the weld joint. The heat capacities for the friction-welded parts welded by rotation speeds of 330 rpm, 490 rpm, 650 rpm, 950 rpm, and 1350 rpm are −1.002 mW/mg, −0.8127 mW/mg, −0.626 4mW/mg, −1.759 mW/mg, and -2.287 mW/mg, respectively. The DSC curve for the friction-welded parts welded by a rotation speed of 1350 rpm shows an endothermic peak [31] between 400 to 440 °C and an exothermic peak [32] between 600 to 700 °C. This means that higher rotation speed contributes to higher molecular orientation in the weld joints, showing that the friction-welded parts have better mechanical properties. Therefore, the structure of the friction-welded parts welded by a rotation speed of 1350 rpm is stronger.

According to the research results, the remarkable findings provide potential industrial values in the polymer-welding industry, since the RFW of dissimilar polymer rods is a green manufacturing method based on four sustainable development goals, i.e., SDG_S_ 7, 8, 9, and 12 [33,34,35,36,37]. In particular, the RFW of polymer rods can be applied for joining the fluid mechanical components, automotive components, axle shafts, or aerospace components [38,39]. In this study, a conventional turning machine was used to carry out the RFW of polymer rods. In future studies, a high-speed lathe or computer numerical control lathe [40,41] are recommended to carry out RFW [42,43,44,45]. These topics are currently being investigated, and the results will be presented in future work.

## 4. Conclusions

The advantages of RFW include efficiency of production, low heat input, and environmental friendliness. PC plastic has very good heat resistance and is widely employed for more robust materials. ABS plastic provides high tensile strength and is very resistant to chemical corrosion and physical impacts. To establish domain knowledge of the RFW of ABS and PC rods, this work reports the joining of FDM-printed dissimilar thermoplastic PC and ABS rods. To establish domain knowledge of the RFW of ABS and PC rods, the RFW experiment was performed using a turning machine. The main conclusions from the experimental work in this study are as follows:

1. The average surface hardness, flexural strength, and impact energy in the weld joint are increased with increasing rotation speed of RFW. The maximum joint strength of 132 MPa, Shore A hardness of 80, and impact energy of 156 J are obtained at a rotation speed of 1350 rpm.
2. The quality of RFW is excellent, since the welding strength in the weld joint is better than that of the ABS base materials. The fracture occurs in the ABS rods since the brittleness of the ABS rods is higher than that of the PC rods.
3. The average percentage error for predicting the peak temperature using COMSOL software using a mesh element count of 875,688 for five different rotation speeds is about 16.6%.
4. The heat capacities for the friction-welded parts welded by rotation speeds of 330 rpm, 490 rpm, 650 rpm, 950 rpm, and 1350 rpm are −1.002 mW/mg, −0.8127 mW/mg, −0.626 4mW/mg, −1.759 mW/mg, and −2.287 mW/mg, respectively. The DSC curve for the friction-welded parts welded by a rotation speed of 1350 rpm shows an endothermic peak between 400 to 440 °C and an exothermic peak between 600 to 700 °C, showing that the friction-welded parts have better mechanical properties.

## Data Availability

Data and materials are available.
